# *SLC26A4* C.317C > A Variant: Functional Analysis and Patient‐Derived Induced Pluripotent Stem Line Development

**DOI:** 10.1002/mgg3.70098

**Published:** 2025-04-22

**Authors:** Yijing Li, Tao Sun, Sang Hu, Hongen Xu, Teng Zhang, Jinlong Liu, Shuangshuang Lu, Bing Wang, Guo Dan

**Affiliations:** ^1^ National Center for International Research in Cell and Gene Therapy, Sino‐British Research Centre for Molecular Oncology, School of Basic Medical Sciences Zhengzhou University Zhengzhou China; ^2^ Department of Clinical Medicine Henan Medical College Zhengzhou China; ^3^ Laboratory of Hearing Loss Mechanism Henan Provincial Medical Key Laboratory Zhengzhou China; ^4^ Precision Medicine Center, Academy of Medical Science, Tianjian Laboratory of Advanced Biomedical Sciences Zhengzhou University Zhengzhou China; ^5^ Department of Obstetrics and Gynecology The Second Affiliated Hospital of Zhengzhou University Zhengzhou China; ^6^ Department of Basic Clinical Medicine Henan Medical College Zhengzhou China

**Keywords:** c.317C > a, hearing loss, iPSCs, *SLC26A4*

## Abstract

**Background:**

*SLC26A4* is the second most common cause of hereditary hearing loss worldwide. This gene predominantly harbors pathogenic variants, including splice, nonsense, and missense. Although missense variants are relatively common, their specific effects on protein function remain unclear. Consequently, there is an urgent need to establish an in vitro system to investigate how these variants impact *SLC26A4* protein function.

**Methods:**

Genetic testing was conducted to determine the specific types of underlying genetic variants in patients. Following this, we employed plasmid transfection to evaluate the effects of the variants on both protein expression levels and the protein's subcellular localization. Thereafter, we transformed peripheral blood mononuclear cells (PBMCs) from the proband into induced pluripotent stem cells (iPSCs) through Sendai virus‐mediated transduction.

**Results:**

Genetic testing revealed that the proband carried compound heterozygous variants: *SLC26A4* c.919‐2A > G and c.317C > A. The c.317C > A variant markedly decreased the expression levels of *SLC26A4* mRNA and its encoded protein. Additionally, it led to the protein's accumulation in the cytoplasm as aggregates. We successfully reprogrammed peripheral blood mononuclear cells from the proband into induced pluripotent stem cells (iPSCs) and verified that these iPSCs retained their pluripotency, differentiation potential, and genetic integrity.

**Conclusion:**

These results provide important insights into the mechanisms by which *SLC26A4* gene variants lead to hearing loss.

## Graphical abstract

We identified compound heterozygous variants in the *SLC26A4* gene, c.919‐2A > G and c.317C > A, and studied how the c.317C > A variant affects Pendrin expression and function. We successfully induced pluripotent stem cells (iPSCs) from peripheral blood mononuclear cells (PBMCs) of the proband.

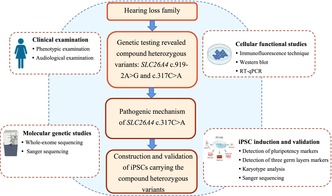

## 1 Introduction

Hearing impairment is one of the most common sensory disorders worldwide, with the prevalence of congenital hearing impairment being approximately 1 in 1000 (Morton and Nance 2006). There are many causes of hearing impairment, and about half of them are attributed to genetic factors (Marazita et al. 1993). Syndromic hearing loss and nonsyndromic hearing loss mimics are common in pediatric patients who initially present with isolated hearing loss (Xiang et al. 2022), highlighting the importance of comprehensive genetic testing. The comprehensive genetic testing and reanalysis of sequencing data provide not only a high diagnostic yield but also valuable information for clinicians to uncover subclinical or pre‐symptomatic phenotypes (Sun et al. 2019; Xiang et al. 2022). Scientists have identified more than 150 deafness‐causing genes to date (http://hereditaryhearingloss.org), with *GJB2*, *SLC26A4* (OMIM#605646), and *MT‐RNR1* being the most common in the world (Du et al. 2014).

*SLC26A4* is the second‐most common cause of hereditary hearing impairment, and it can cause autosomal recessive Pendred syndrome and autosomal recessive deafness type 4 with enlarged vestibular aqueduct (EVA) (Wang et al. 2019). Different proportions of patients having biallelic *SLC26A4* mutations were revealed in a relatively limited number of large NSHL studies performed without preselection of patients with EVA or Pendred syndrome: 3.5% of the Europe and the Americas child population, 0.9% of Europe patients, 2.9% of South America patients, 6.3% (0%–8.3%) of patients from West Asia, 10.7% of patients from South Asia, and 23.2% ~ 23.7% of East Asia patients (Danilchenko et al. 2021). The *SLC26A4* gene has 21 exons encoding the multiple transmembrane protein Pendrin, which consists of 780 amino acids. Primarily expressed in the inner ear, thyroid, and kidney, Pendrin serves as a monoanionic exchanger, mediating the transport of anions like Cl^−^, I^−^, OH^−^, and HCO_3_^−^ (Doerfer et al. 2020). The diseases caused by *SLC26A4* are inherited in an autosomal recessive manner, with both homozygous and compound heterozygous pathogenic variants capable of causing the disease. The pathogenic variants of *SLC26A4* primarily include nonsense, splice, and missense variants. Nonsense variants typically lead to loss of function, while splice variants can be functionally determined through experimental methods such as minigenes. However, the functional consequences of many missense variants remain unclear, highlighting the need to establish an in vitro functional system to explore the impact of these variants on pendrin function .

Induced pluripotent stem cells (iPSCs) are cells that are reprogrammed from terminally differentiated somatic cells into pluripotent stem cells by introducing specific transcription factors. These cells share similarities with human embryonic stem cells (hESCs) in terms of gene expression patterns, surface proteins, and differentiation capacities (Patel and Yang 2010). Compared to hESCs, iPSCs offer several significant advantages: (1) Ethical and Moral Considerations: iPSCs can be derived from the patient's own somatic cells, thereby avoiding the ethical and moral dilemmas associated with the use of human embryos. (2) Low Immunogenicity: Since iPSCs originate from the patient's somatic cells, they carry the same genetic information, resulting in lower immunogenicity and reduced risk of immune rejection following transplantation. Additionally, iPSCs technology provides valuable in vitro models for disease research (Kawamura et al. 2012), drug testing (Xu and Zhong 2013), and gene therapy evaluation (Devine et al. 2011). Therefore, iPSC‐derived inner ear organoids can be used for large‐scale preliminary screening of hearing rescue drugs and to evaluate emerging therapies such as cell therapy and gene therapy (Zong et al. 2024).

In this study, we identified a new family with recessive hereditary hearing loss with an enlarged vestibular aqueduct. To determine the causative genes and variants in this family, we utilized a hearing loss gene detection kit based on multiplex PCR and next‐generation sequencing (NGS), which we developed in our laboratory (Tian et al. 2021). The results revealed that the affected individuals carried compound heterozygous variants of the *SLC26A4* gene, specifically c.919‐2A > G and c.317C > A. Without conducting functional studies, based on the American College of Genetics and Genomics (ACMG) guidelines for variant interpretation, this variant can only be categorized as a Variant of Uncertain Significance (VUS), which is supported by the criteria of PM3, PM2_supporting, and PP4. To investigate whether the *SLC26A4* c.317C > A variant affects the expression and function of Pendrin, we assessed its effects on mRNA, protein expression levels, and the localization of Pendrin proteins in a cell model. Additionally, we extracted peripheral blood mononuclear cells (PBMCs) from the affected patients and reprogrammed these PBMCs into iPSCs using Sendai virus transfection. The pluripotency of the resulting cells was confirmed through RT‐qPCR and immunofluorescence, and the differentiation potential was confirmed using an in vitro three‐germ‐layer differentiation assay. Furthermore, the genetic integrity of the cell line was validated through karyotyping, Short Tandem Repeat (STR) analysis, and Sanger sequencing. These findings lay a crucial foundation for a deeper understanding of the mechanisms underlying hearing loss caused by *SLC26A4* variants.

## 2 Materials and Methods

### 2.1 Ethical Compliance

This study follows the ethical guidelines of the Helsinki Declaration, and the research protocol has received approval from the Ethics Committee of The Second Affiliated Hospital of Zhengzhou University (approval number: 2018008). Written informed consent was obtained from all participating individuals or their guardians prior to enrollment in the study.

### 2.2 Subject Recruitment and Clinical Evaluation

The subjects in this study were patients with hearing loss and their families who attended the Second Affiliated Hospital of Zhengzhou University. We did a full clinical evaluation of the subjects, which included hearing tests like tympanometry, otoscopy, pure tone audiometry, distortion product otoacoustic emission (DPOAE), auditory brainstem response (ABR), auditory steady‐state response (ASSR), and 40 Hz auditory event‐related potentials (AERP) test. In addition, the subjects underwent a high‐resolution temporal bone CT (computerized tomography) scan. The results of axial CT scanning led to the diagnosis of EVA in the patients, defined as a canal width exceeding 1.5 mm between the common vestibular peduncle and the midpoint between the outer orifices of the vestibular aqueducts.

### 2.3 Genetic Testing and Bioinformatics Analysis

We collected 2 mL of venous peripheral blood from each of our subjects (with EDTA as the anticoagulant) and extracted genomic DNA using a whole‐blood genomic DNA extraction kit (GenMagBio, NA001). Our laboratory developed in‐house kits to detect common hearing loss genes, as previously described in the literature (Tian et al. 2021). We conducted a bioinformatic analysis of high‐throughput sequencing data, followed by variant annotation and filtering. Subsequently, we assessed the pathogenicity of the suspected variants, referencing American College of Medical Genetics and Genomics(ACMG) guidelines (Richards et al. 2015) and the expert specification of the ACMG/AMP variant interpretation guidelines for genetic hearing loss by the ClinGen Hearing Loss Expert Panel (Oza et al. 2018). For the identified suspected pathogenic variants, we performed Sanger sequencing (Sangon Biotech, Shanghai) to verify the accuracy and reliability of our results.

### 2.4 Vector Construction and Cell Transfection

We used the plasmid pIRES‐EYFP‐*SLC26A4*, which contains the open reading frame (ORF) of the wild‐type *SLC26A4* gene as a template (constructed by Xitubio), and amplified the *SLC26A4* ORF fragment using the primer *SLC26A4*‐FLAG (see Table 1) with DNA Polymerase (TAKARA's PrimeSTAR Max DNA Polymerase). After that, we performed restriction digestion using MluI‐HF (NEB, #R3198) and NheI‐HF (NEB, #R3131). Then, we used T4 DNA ligase (Thermo, EL0011) for ligation to construct the wild‐type *SLC26A4* expression plasmid pIRES‐EYFP‐SLC26A4(WT)‐FLAG, which has a Flag tag fused at the C‐terminus of *SLC26A4* for subsequent immunofluorescence and Western blot experiments. To investigate the effect of the *SLC26A4* c.317C > A variant, we constructed the expression vector pIRES‐EYFP‐SLC26A4(MUT)‐FLAG for the *SLC26A4* c.317C > A variant using a targeted mutagenesis kit (Vazyme, C214). We introduced the *SLC26A4* c.317C > A variant using the primer *SLC26A4*‐MUT, as listed in Table 1, resulting in the construction of the pIRES‐EYFP‐*SLC26A4*(MUT)‐FLAG vector. The reference sequence for the *SLC26A4* gene used in this study is NM_001126112.3.

**TABLE 1:** RT‐qPCR and PCR reaction primer sequences.

Gene	Primer sequence
NANOG‐F	CTGCAGAGAAGAGTGTCGCA
NANOG‐R	CCAGGTCTTCACCTGTTTGT
DPPA4‐F	GCTCCAAAGGCCAGAAATTG
DPPA4‐R	AACTTTGCAGGGACGTTTCC
SOX2‐F	GCTACAGCATGATGCAGGACCA
SOX2‐R	TCTGCGAGCTGGTCATGGAGTT
OCT4‐F	CGACCATCTGCCGCTTTG
OCT4‐R	GCCGCAGCTTACACATGTTCT
GAPDH‐F	TCGGAGTCAACGGATTTGGT
GAPDH‐R	TTCCCGTTCTCAGCCTTGAC
SLC26A4‐F^a^	TGGTGGGATCTGTTGTTCTGA
SLC26A4‐R	TGGTGGGATCTGTTGTTCTGA
SLC26A4‐mut‐F	GGCATATGACCTACTAGCTGCAGTTCCTGTCG
SLC26A4‐mut‐R	CTAGTAGGTCATATGCCATCCCTTGCAGCGTG
SLC26A4‐FLAG‐F	ATACGACTCACTATAGGCTAGGCTAGCCACCATGGCAGCGCCAGGC
SLC26A4‐FLAG‐R	CTAGATGCATGCTCGACGCGTGAATTCTCACTTGTCA TCGTCGTCCTTGTAATCGGATGCAAGTGTACG

^a^ Reference sequence: NM_000441.2.

We cultured human renal embryonic HEK293 cells and HeLa cells (both from the Chinese Academy of Sciences cell bank) in DMEM (Gibco, C11995500BT) containing 10% FBS (Clark Bioscience, FB25015) and grew the cells at 37°C and 5% CO_2_. After passage culture, we inoculated HEK293 and HeLa cells into six‐well and four‐chamber cell culture plates, respectively, and allowed them to grow overnight to approximately 50% confluence. Next, we transfected 1 μg of plasmid per well using a 2:1 ratio of plasmid to transfection reagent PEI (Servicebio, G1802). Within 8 h of transfection, we switched the medium to a complete one, and 48 h later, we extracted the total RNA and protein from HEK293 cells using an RNA extraction kit (SparkJade, AC0202) and a RIPA strong lysate (Servicebio, G2002) that contained 1% 100 mM PMSF (Servicebio, G2008), respectively, for real‐time quantitative Polymerase Chain Reaction (RT‐qPCR) and immunoblotting experiments. We simultaneously used the transfected HeLa cells for immunofluorescence experiments.

### 2.5 RT‐qPCR and Western Blotting

Total RNA was reverse transcribed into cDNA using HiScript III All‐in‐one RT SuperMix for qPCR (Vazyme, R323). Subsequently, qPCR was performed using the primers *SLC26A4*‐QF/QR and ChamQ Universal SYBR qPCR Master Mix (Vazyme, Q711). Each sample was analyzed in triplicate for expression using the $2^{-\Delta \Delta C_{T}}$ method and normalized to the endogenous control ACTIN mRNA. The primers used are listed in Table S1.

Protein concentrations were calculated using the BCA Protein Assay Kit. SDS‐PEAG electrophoresis was performed on 15 μg of each protein with 10% SDS‐PAEG gels (CWBIO, CW0022S), then the proteins were transferred to polyvinylidene difluoride (PVDF) membranes (Millipore, IPVH0010), and incubated with primary antibodies against β‐actin Rabbit Polyclonal Antibody (HUABIO, R1102, 1:1000), Flag‐Tag Mouse Monoclonal Antibody (Abways, AB0008, 1:5000) at 4°C overnight. After washing three times with 1 × TBST, the membranes were incubated with a secondary antibody, HRP Conjugated Goat anti‐rabbit IgG Polyclonal Antibody (HUABIO, HA1001, 1:80000), or HRP Conjugated Goat anti‐mouse IgG Polyclonal Antibody (HUABIO, HA1006, 1:50000) for 1 h at room temperature. After washing, the membrane and the Immunoreactive bands were imaged with ECL luminous fluid (Servicebio, G2014).

### 2.6 Immunofluorescence

Cells from adherent cultures were fixed with 4% paraformaldehyde in 1 × PBS for 15 min and permeabilized with 0.5% Triton X‐100 in 1 × PBS for 30 min at room temperature. Then, the cells were washed twice with 1 × PBS and blocked with 5% BSA in 1 × PBS for 30 min. Following this, they were incubated overnight at 4°C with the Flag antibody (Abways, AB0008, 1:100) and the Pan‐Cadherin antibody(HUABIO, ET1609‐70, 1:100), and hatched using secondary antibodies CoraLite488‐conjugated Donkey Anti‐MOUSE (HUABIO, HA1125, 1:500) for 1 h at room temperature in the dark after washing with 1 × PBS. Cell nuclei were probed with 4',6‐diamidino‐2‐phenylindole Dihydrochlorides (DAPI, Servicebio, G1047). The Fluorescence confocal images were obtained with a confocal microscope (Zeiss, LSM880).

### 2.7 Human iPSCs Reprogramming and Culture

PBMCs from the proband were isolated by density gradient centrifugation using Ficoll (TBD, LTS1077) and were maintained in Stempro‐34 SFM completed medium (Gibco, 10,639,011) supplemented with 100 ng/mL FLT3 (Gibco, GMP300‐19‐1MG), 100 ng/mL SCF (Cell Signaling, 87318S), 20 ng/mL IL3 (Cell Signaling, 8918) and 20 ng/mL IL6 (Cell Signaling, 48,333) for 4 days. Then, 3 × 10^5^ cells/well of the obtained PBMC were transferred to a 24‐well plate and reprogrammed using CytoTune‐iPS 2.0 Sendai Reprogramming Kit (Thermo Fisher Scientific, A16517) according to the manufacturer's protocol. The Sendai viruses were removed by centrifugation the next day. Then, the culture medium was replaced with Essential 8 Medium (Gibco, A1517001), and the culture medium was changed daily. Induced pluripotent stem cells are incubated at 37°C in a humidified atmosphere of 5% CO_2_. Until 15–21 days after transduction, the Colonies were picked onto vitronectin‐coated 6‐well culture plates for expansion.

### 2.8 The Pluripotency Identification of Human iPSCs by Immunofluorescence

The antibodies OCT4 (abcam, ab181557, 1:200), NANOG (abcam, ab109250, 1:200), SOX2 (abcam, ab92494, 1:200), and SSEA4 (abcam, ab16287, 1:200) were used to detect the pluripotency of iPSCs by immunofluorescence as described previously. The secondary antibodies used were iFluor488 Conjugated Goat anti‐rabbit IgG Polyclonal Antibody (HUABIO, HA1121, 1:500) and iFluor 594 Conjugated Goat anti‐rabbit IgG Polyclonal Antibody (HUABIO, HA1122, 1:500). The Fluorescence confocal images were obtained with a confocal microscope (Zeiss, LSM880).

### 2.9 In Vitro Three Germ Layer Differentiation

The in vitro differentiation potential of iPSCs was detected using the three germ layers' formation assay. According to the STEMdiff Trilineage Differentiation Kit (STEMCELL Technologies, 05230) protocol, the cells were cultured to days 5 and 7 for staining, respectively. Immunofluorescence staining specific for ectodermal βIII‐Tubulin (Abcam, ab7751, 1:500) mesodermal α‐SMA (Abcam, ab7817, 1:500) and endodermal AFP (Proteintech, 14,550‐1‐AP, 1:500) markers.

### 2.10 Karyotyping and Mutation Analysis

To characterize the chromosomal abnormality of iPSCs, the iPSC cell lines were karyotypically assessed at generation 20 at KingMed Center for Clinical Laboratory (Zhengzhou, China) using standard protocols for high‐resolution G banding. Genomic DNA extracted from iPSCs was performed as described for the Peripheral blood samples. The genomic DNA of the iPSCs served as a template for PCR amplification using the primers listed in Table 1, and Sanger sequencing analysis was subsequently performed.

## 3 Results

### 3.1 Genetic Diagnosis of Patients With Hearing Loss Identified a Novel Rare *SLC26A4* Variant c.317C > A

**FIGURE 1:**
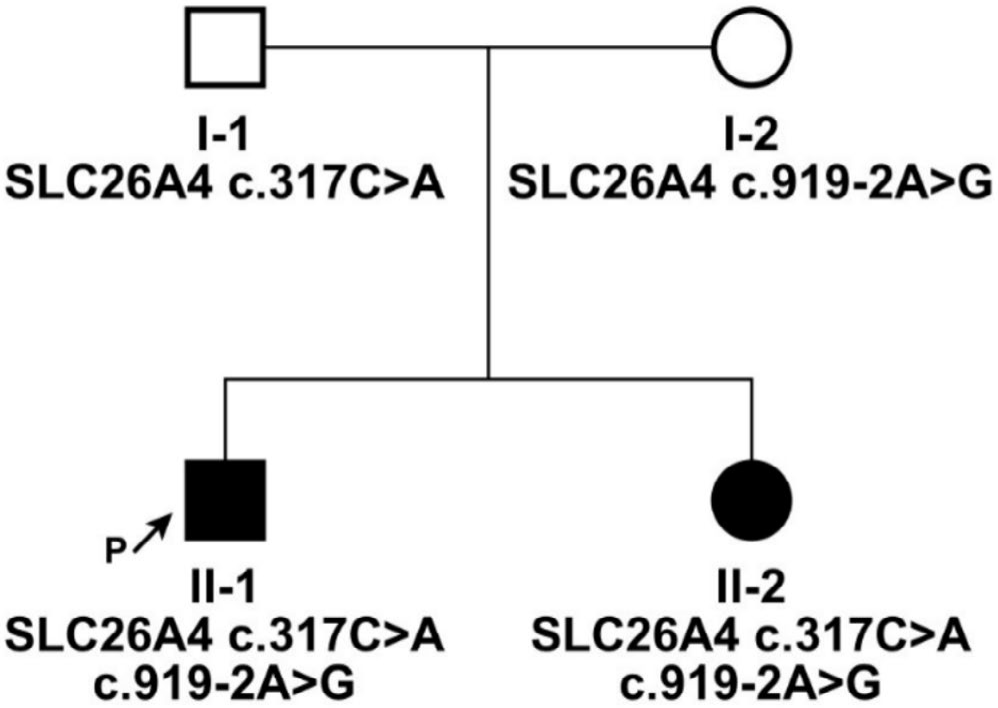
Family pedigree of the hearing loss patients combined with EVA. The family extends across two generations, with two individuals diagnosed with compound heterozygous variants *SLC26A4* c.317C > A and c.919‐2A > G. Females are denoted by circles, while males are indicated by squares.

We identified a new family with hereditary hearing loss during our molecular diagnostics for patients with hearing loss, consisting of two siblings who are both deaf. Initially, we conducted a systematic clinical evaluation and audiometric testing on the proband and his sister, which revealed that both had profound hearing loss accompanied by enlarged vestibular aqueducts. To further explore the pathogenic genes and variants within this family, we utilized a laboratory‐developed kit based on multiplex PCR and NGS technology specifically designed for detecting pathogenic variants in common hearing loss genes for Chinese (Tian et al. 2021). Our analysis revealed that both the proband and his sister carried compound heterozygous variants in the *SLC26A4* gene: c.919‐2A > G and c.317C > A (Figure 1). PCR and Sanger validation of the variants were performed on all family members, revealing that c.919‐2A > G was inherited from the mother and c.317C > A from the father. The former is a common pathogenic variant, while the latter is a rare variant. According to the ACMG guidelines, the *SLC26A4* c.317C > A variant is classified as VUS (PM3 + PM2_supporting+PP4).

### 3.2 The Variant c.317C > A Changed the Expression and Localization of Pendrin

**FIGURE 2:**
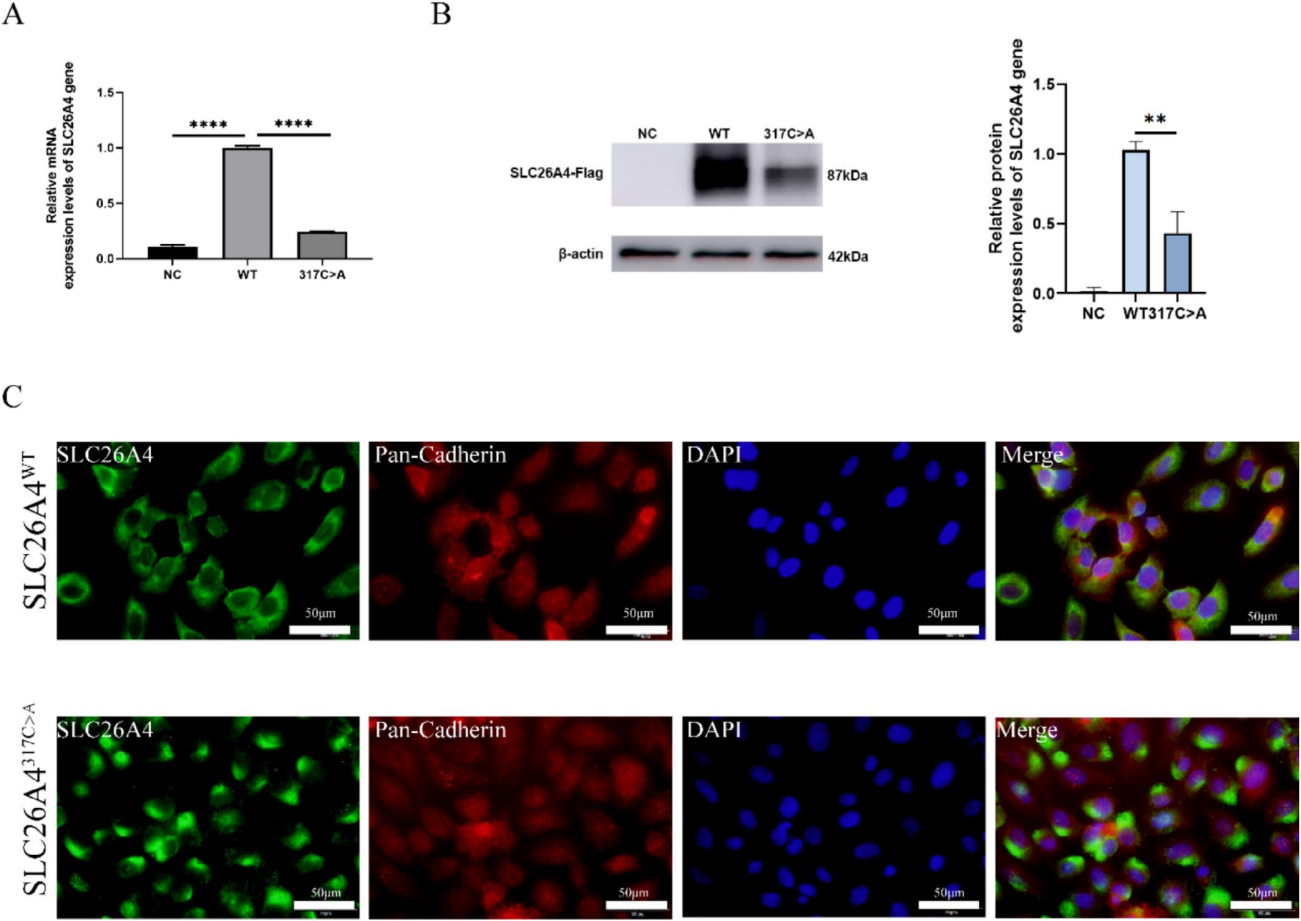
The effect of c.317C > A variant on the expression of the *SLC26A4* gene. (A) Relative expression levels of wild‐type (WT) and c.317C > A variant mRNA in HEK293 cells as determined by RT‐qPCR (*n* = 3). (B) Protein expression levels of the wild‐type (WT) and c.317C > A variant as determined by Western blot. (The results are shown as the mean ± standard deviation (SD). ns, not significant, **p < 0.01, ****p < 0.0001, unpaired, two‐tailed Student's *t* test, with all comparisons normalized to the WT group.) (C). Protein localization analysis of the wild‐type (WT) and c.317C > A variant assessed by Immunofluorescence. HeLa cells were transfected with plasmids encoding both wild‐type and variant and subsequently fixed and stained with DAPI to visualize cell nuclei and facilitate quantification of cell density. Immunostaining for Pan‐Cadherin (red) and *SLC26A4* (green). The scale bar represents 50 μm.

In this study, we transiently expressed the human *SLC26A4* gene in HEK293 cells to investigate the differences in expression levels between the wild type and the c.317C > A variant. RT‐qPCR analysis showed that the mRNA levels of the *SLC26A4* gene with the c.317C > A variant were significantly lower than those of the wild type (Figure 2A), suggesting that this variant may affect transcription or mRNA stability of *SLC26A4*. Additionally, Western blot analysis was performed to assess the expression level of Pendrin in total cell membrane proteins, which indicated that the expression of the Pendrin protein with the c.317C > A variant was significantly reduced (Figure 2B and Figure S1). This decrease in protein levels may be attributed to alterations in either protein production or degradation pathways.

To further investigate the subcellular localization of Pendrin, we expressed the human wild‐type and mutant pIRES‐EYFP‐*SLC26A4*‐FLAG in HeLa cells to investigate the distribution of Pendrin within the cells. Immunofluorescence results show wild‐type Pendrin predominantly localized to the plasma membrane, indicating its expected cellular distribution (Figure 2C). Importantly, the C‐terminal FLAG tag did not interfere with Pendrin's transport function. In contrast, the mutated Pendrin displayed abnormal localization patterns. It failed to efficiently target the plasma membrane, resulting in some protein being retained in the cytoplasm and an uneven distribution at the plasma membrane, characterized by conspicuous aggregation (Figure 2C). This aggregation may compromise Pendrin's functionality and, consequently, its physiological roles within the cell.

In summary, wild‐type Pendrin shows preferential transport to the plasma membrane, maintaining normal cellular localization, while the mutated Pendrin of c.317C > A exhibits retention in the cytoplasm and aggregation, suggesting that this variant may lead to a loss or reduction of Pendrin function, potentially playing an important role in related physiological and pathological processes. Upon the inclusion of the PS3 criterion in the evaluation, the *SLC26A4* c.317C > A variant is classified as “Likely Pathogenic”.

### 3.3 Establishment of iPSCs

**FIGURE 3:**
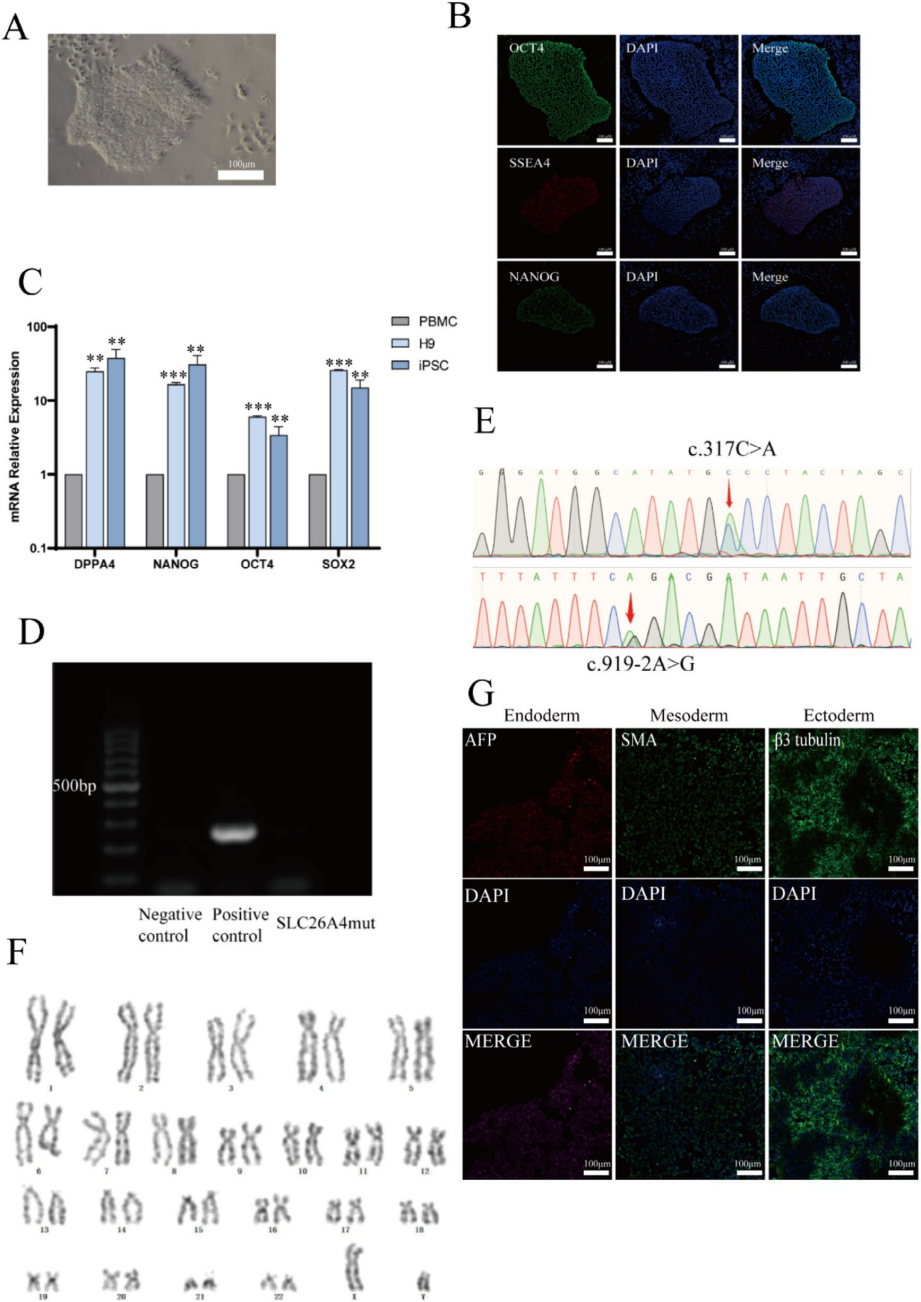
The induction and identification of induced pluripotent stem cells (iPSCs) from the patient‐derived peripheral blood mononuclear cells. (A) Morphology of *SLC26A4* ^mut^‐iPS cells. (B) Detection of pluripotency markers OCT4, SSEA4, and NANOG in *SLC26A4* ^mut^‐iPS cells using immunofluorescence technology. (C) The mRNA expression levels of SOX2, NANOG, OCT4, and DPPA4 in *SLC26A4* ^mut^‐iPS as determined by RT‐qPCR. In the experiment, H9 embryonic stem cells serve as a positive control, and PBMCs serve as a negative control. (GraphPad software was used to analyze the results of three independent experiments, ns, not significant, **p < 0.01, ***p < 0.0001, unpaired, two‐tailed Student's *t* test). (D) Mycoplasma detection for *SLC26A4* ^mut^‐iPS (positive control is the sample provided in the kit, and negative control is deionized water). (E, F) Karyotype analysis and genotyping of *SLC26A4* ^mut^‐ iPS. (G) Detection of the three germ layers' markers AFP, α‐SMA, and β3 tubulin in *SLC26A4* ^mut^‐iPS cells using immunofluorescence technology. The pluripotent differentiation ability of *SLC26A4* ^mut^‐iPS was detected using immunofluorescence technology, with AFP as a marker for the endoderm, α‐SMA as a marker for the mesoderm, and β3 tubulin as a marker for the ectoderm.

By establishing a patient‐derived iPSCs disease model, this study aims to provide novel insights into the pathogenic mechanisms associated with variants in the *SLC26A4* gene. PBMCs were isolated from the proband's peripheral blood. Following 12 days of viral transduction, we observed the emergence of small cell colonies. By day 18, these colonies expanded and became more compact, resulting in the formation of distinct individual cell colonies (Figure 3A). This stage of cell aggregation serves as a crucial foundation for subsequent assessments of pluripotency and differentiation. Ultimately, the iPSC cell line was successfully established.

To validate the pluripotency of the iPSCs, we assessed the transcriptional levels of endogenous pluripotent genes *OCT4*, *SOX2*, *DPPA4*, and *NANOG* in the *SLC26A4* mutant iPSCs (*SLC26A4* ^mut^‐iPS). The results from RT‐qPCR demonstrated varying degrees of upregulation in the expression of these pluripotency genes compared to the negative control (Figure 3C). In the experiment, H9 embryonic stem cells serve as a positive control, and PBMCs serve as a negative control. At the protein level, we selected three stem cell‐specific pluripotency markers—OCT4, SSEA4, and NANOG—and performed immunofluorescence staining on the cultured cells. The staining results demonstrated that *SLC26A4* ^mut^‐iPS exhibited positive expression for all three markers. Notably, nuclear OCT4 and NANOG staining completely overlapped with DAPI‐stained cell nuclei, while SSEA4 exhibited a distribution around the nuclear periphery (Figure 3B). These findings suggest that *SLC26A4* ^mut^‐iPS not only demonstrates pluripotency at the genetic level but also possesses corresponding characteristics at the protein level.

Subsequently, we conducted assessments of the genetic material and karyotype of the induced iPSCs to confirm their freedom from mycoplasma contamination and to ensure that their genetic integrity remained intact. Initially, a mycoplasma test was performed, and the results from agarose gel electrophoresis indicated that *SLC26A4* ^mut^‐iPS tested negative for mycoplasma (Figure 3D), confirming the absence of contamination in this cell line. Following this, we verified the variants present in the cell line. Sanger sequencing results confirmed that the induced stem cells retained the c.317C > A heterozygous variant (Figure 3E), which is consistent with the one identified in the parental PBMCs, thereby demonstrating that the reprogrammed cells preserved the original variant. Lastly, to ensure that the culture of human mononuclear cells during the reprogramming process did not introduce chromosomal or genetic alterations, we performed a karyotype analysis. For the iPSCs at passage 15, we analyzed 16 metaphases, revealing a normal karyotype consisting of 46 chromosomes (Figure 3F) and no evidence of chromosomal structural or numerical abnormalities. Collectively, these results support the feasibility and reliability of *SLC26A4* ^mut^‐iPS as a research model, thereby laying a solid foundation for further exploration of the biological functions associated with the *SLC26A4* mutation. Collectively, these results support the feasibility and reliability of *SLC26A4* ^mut^‐iPS as a research model, thereby laying a solid foundation for further exploration of the biological functions associated with the *SLC26A4* variant.

The trilineage differentiation assay is a crucial indicator for assessing the pluripotency of iPSCs. Immunofluorescence staining results from the trilineage differentiation demonstrated that *SLC26A4* ^mut^‐iPS specifically expressed markers for all three germ layers (Figure 3G), further confirming its potential for multidirectional differentiation.

By establishing a patient‐derived iPSCs disease model, we have opened a new perspective for studying the pathogenic mechanisms of *SLC26A4* gene variants. This work will provide a valuable research model for better elucidating the pathogenic mechanisms of *SLC26A4* in hereditary deafness.

## 4 Discussion

In this study, during the genetic screening and molecular diagnosis of hearing loss patients, we identified a family with two siblings, both affected by hearing loss. Clinical assessment and audiological testing indicated that both siblings exhibited typical bilateral enlargement of the vestibular aqueduct. Using multiplex PCR and NGS technology developed in our laboratory, we detected compound variants in the *SLC26A4* gene—c.317C > A and c.919‐2A > G—in the proband and his sister. The c.317C > A variant was inherited from the father, while the c.919‐2A > G was inherited from the mother. To further explore the pathogenic mechanism of the rare variant c.317C > A, we analyzed the impact of this variant on the expression and localization of the protein product. The results showed that the expression of the mutated protein was reduced, and it was mainly distributed in a punctate pattern in the cytoplasm. Additionally, we extracted PBMCs from the patients and successfully induced them into disease‐specific iPSCs. The induction results confirmed the successful establishment of the iPSCs disease model, retaining the variant site and maintaining an unchanged karyotype. This study clarifies the effects of the rare variant c.317C > A on protein expression and subcellular localization. This finding broadens our understanding of the mutational landscape of pathogenic variations within the *SLC26A4* gene. Moreover, the iPSCs disease model we have developed serves as a potent resource for future studies aimed at unraveling the pathogenic mechanisms associated with this pathogenic variant.

Pathogenic variants in the *SLC26A4* gene are the main cause of enlarged vestibular aqueducts (Zhao et al. 2024). The most common two causative variants of *SLC26A4* were c.919–2 A > G and c.2168 G > A (Wu et al. 2022). Although some studies have suggested that *KCNJ10* and *FOXI1* may also be associated with EVA, this view remains controversial (Klarov et al. 2022; Landa et al. 2013). Pathogenic variants in the coding region or splice sites of *SLC26A4* in the patients can be categorized into several genotypes: homozygous or compound heterozygous variants are referred to as M2, those with no detected *SLC26A4* mutations are called M0, and those with only one allelic variant are designated as M1. Studies have indicated that the occurrence of M2 is relatively low in non‐Hispanic whites, accounting for only 25% (Honda and Griffith 2022). In contrast, in East Asian populations, including China, Korea, and Japan, the prevalence of M2 is significantly higher, reaching 67%–90% (Honda and Griffith 2022). Previous research indicates that the M2 category encompasses a subset of compound heterozygous variants, with one allele often being a prevalent variant (i.e., c.919‐2A > G) and the other being a less common one (Wang et al. 2007). This highlights the importance of screening for rare variants in the *SLC26A4* gene.

Pendrin is an anion exchange protein located on the plasma membrane, capable of mediating the transport of chloride, iodide, bicarbonate, and formate (Yoon et al. 2008). Pendrin was synthesized by ribosomes, transported from the endoplasmic reticulum to the Golgi apparatus via transport vesicles, and then anchored to the plasma membrane (Gee et al. 2018; Rauter et al. 2020). By examining the levels of Pendrin in the membrane and cytoplasm, we discovered that the expression level of the mutant Pendrin (p. A106D) was significantly lower than that of the wild‐type Pendrin. This is consistent with the effects of other missense variants, such as p.L236P, p.T416P, p.G384E, p.E29K, p.D669V, and p.V285I, p.L117F, p.E29K, P.D669V, p.V285I, p.H723R/p.R677W, and p.R677W on Pendrin expression (Dai et al. 2022; Matulevicius et al. 2022; Rotman‐Pikielny et al. 2002). In vitro cellular assays focused on the p.L445W and p.M147T variants within Pendrin have demonstrated that these missense variants impede the proper translocation of Pendrin to the plasma membrane (Rebeh et al. 2010). This could be a key mechanism by which the missense variants in the *SLC26A4* gene lead to related diseases (Rotman‐Pikielny et al. 2002). In the immunofluorescence experiments, we found that the mutant Pendrin p. A106D exhibited uneven distribution and punctate patterns in the cytoplasm. This aligns with the research conducted by Makoto Hosoya and colleagues on p.M147V, p.H723K, and p.T410M (Hosoya et al. 2017). Their study observed that the cytoplasmic aggregates frequently co‐localized with ubiquitin and microtubule‐associated protein 1 light chain 3 beta (LC3b). This suggests that both the ubiquitin‐proteasome system and autophagy pathways may play a role in the degradation of intracellular aggregates formed by Pendrin mutants.

Additionally, we have successfully established an iPSCs model derived from a patient carrying compound heterozygous variants c.317C > A and c.919‐2A > G in the *SLC26A4* gene. Before this, there were only iPSCs models derived from patients with homozygous variants c.919‐2A > G, c.1229C > T, c.2168A > G, and compound heterozygous variants c.439A > G and c.2168A > G (Cheng et al. 2019; Hosoya et al. 2017). In this study, the reprogrammed iPSCs did not exhibit any chromosomal abnormalities after multiple passages. In our forthcoming research, we aim to differentiate the iPSCs into cochlear cell models that reflect the specific disease context. This approach will offer a more pertinent platform for delving into the pathogenic mechanisms associated with this genetic variant and pave the way for future drug screening endeavours.

In conclusion, our study confirms the pathogenicity of the novel *SLC26A4* gene variant c.317C > A. The mutated Pendrin protein exhibits significantly reduced expression levels when compared to the wild‐type protein. Additionally, while the wild‐type Pendrin is directed to the plasma membrane, the mutated form is partially retained within the cytoplasm, forming distinct punctate aggregates. Moreover, we have successfully established a robust iPSCs disease model, which provides a solid foundation for future comprehensive investigations into the role of the *SLC26A4* gene in hereditary hearing loss.

## Author Contributions

Research design: Bing Wang and Dan Guo. Patient phenotype analysis and genetic counseling: Tao Sun, Bing Wang, Dan Guo. Targeted Sequencing, Sanger Sequencing, and variant Interpretation: Tao Sun, Hongen Xu, Bing Wang. Somatic Cell Verification and iPSCs Induction: Yijing Li, Sang Hu, Jinlong Liu, Shuangshuang Lu. Writing and Reviewing Manuscript Drafts: Yijing Li, Tao Sun, Teng Zhang, Bing Wang, Dan Guo. All authors have reviewed and approved the final version of the manuscript.

## Ethics Statement

This study follows the ethical guidelines of the Helsinki Declaration, and the research protocol has received approval from the Ethics Committee of The Second Affiliated Hospital of Zhengzhou University (approval number: 2018008).

## Consent

Written informed consent was obtained from all participating individuals or their guardians prior to enrollment in the study.

## Conflicts of Interest

The authors declare no conflicts of interest.

## Supporting information


Figure S1.

Table S1.

## Data Availability

The data that support the findings of this study are available from the corresponding author upon reasonable request.
