# Supplementary figures and images for: SLC26A4 C.317C > A Variant: Functional Analysis and Patient‐Derived Induced Pluripotent Stem Line Development
Source: Mol Genet Genomic Med. 2025 Apr 22;13(4):e70098. doi: 10.1002/mgg3.70098 (PMC12012755; doi:10.1002/mgg3.70098)

**Figure S1.** The raw images of the Western blot


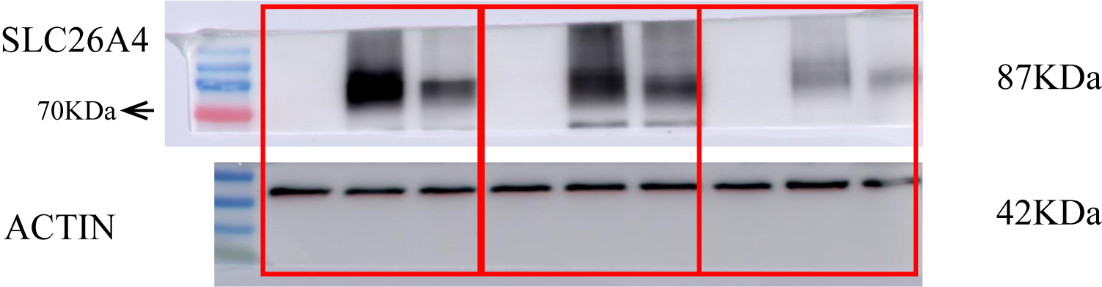

Supplement: Supplementary file 1 — Figure S1. [file MGG3-13-e70098-s001.docx]
