# Supplementary material for: SLC26A4 C.317C > A Variant: Functional Analysis and Patient‐Derived Induced Pluripotent Stem Line Development
Source: Mol Genet Genomic Med. 2025 Apr 22;13(4):e70098. doi: 10.1002/mgg3.70098 (PMC12012755; doi:10.1002/mgg3.70098)
Supplement: Supplementary file 2 — Table S1. [file MGG3-13-e70098-s002.docx]

Supplementary Table S1 Primers used in this study.

| Primer name | Primer sequence |
| --- | --- |
| NANOG-F | CTGCAGAGAAGAGTGTCGCA |
| NANOG-R | CCAGGTCTTCACCTGTTTGT |
| DPPA4-F | GCTCCAAAGGCCAGAAATTG |
| DPPA4-R | AACTTTGCAGGGACGTTTCC |
| SOX2-F | GCTACAGCATGATGCAGGACCA |
| SOX2-R | TCTGCGAGCTGGTCATGGAGTT |
| OCT4-F | CGACCATCTGCCGCTTTG |
| OCT4-R | GCCGCAGCTTACACATGTTCT |
| GAPDH-F | TCGGAGTCAACGGATTTGGT |
| GAPDH-R | TTCCCGTTCTCAGCCTTGAC |
| SLC26A4-F | TGGTGGGATCTGTTGTTCTGA |
| SLC26A4-R | TGGTGGGATCTGTTGTTCTGA |
| SLC26A4-mut-F | GGCATATGACCTACTAGCTGCAGTTCCTGTCG |
| SLC26A4-mut-R | CTAGTAGGTCATATGCCATCCCTTGCAGCGTG |
| SLC26A4-FLAG-F | ATACGACTCACTATAGGCTAGGCTAGCCACCATGGCAGCGCCAGGC |
| SLC26A4-FLAG-R | CTAGATGCATGCTCGACGCGTGAATTCTCACTTGTCATCGTCGTCCTTGTAATCGGATGCAAGTGTACG |
